# Using temporal detrending to observe the spatial correlation of traffic

**DOI:** 10.1371/journal.pone.0176853

**Published:** 2017-05-04

**Authors:** Alireza Ermagun, Snigdhansu Chatterjee, David Levinson

**Affiliations:** 1 Department of Civil and Environmental Engineering, Northwestern University, Evanston, Illinois, United States of America; 2 School of Statistics, University of Minnesota, Minneapolis, Minnesota, United States of America; 3 School of Civil Engineering, University of Sydney, Sydney, New South Wales, Australia; Beihang University, CHINA

## Abstract

This empirical study sheds light on the spatial correlation of traffic links under different traffic regimes. We mimic the behavior of real traffic by pinpointing the spatial correlation between 140 freeway traffic links in a major sub-network of the Minneapolis—St. Paul freeway system with a grid-like network topology. This topology enables us to juxtapose the positive and negative correlation between links, which has been overlooked in short-term traffic forecasting models. To accurately and reliably measure the correlation between traffic links, we develop an algorithm that eliminates temporal trends in three dimensions: (1) hourly dimension, (2) weekly dimension, and (3) system dimension for each link. The spatial correlation of traffic links exhibits a stronger negative correlation in rush hours, when congestion affects route choice. Although this correlation occurs mostly in parallel links, it is also observed upstream, where travelers receive information and are able to switch to substitute paths. Irrespective of the time-of-day and day-of-week, a strong positive correlation is witnessed between upstream and downstream links. This correlation is stronger in uncongested regimes, as traffic flow passes through consecutive links more quickly and there is no congestion effect to shift or stall traffic. The extracted spatial correlation structure can augment the accuracy of short-term traffic forecasting models.

## Introduction

The rapid development of technology and new availability of large amounts of data enhance the ability to monitor and forecast mobility and, more specifically, traffic over time and space [1–4]. Traffic analysts have utilized the spatial dependency of road segments to solve three typical problems in a traffic network: (1) short-term traffic forecasting [1, 2, 5, 6], (2) reliable path problem [7], and (3) missing data estimation [8].

Irrespective of the problem, studies have revealed the positive spatial correlation between road segments for two main reasons. First, the topology of studied networks typically consists of traffic links that are immediately upstream or downstream, and thereby they exhibit positive correlation in terms of traffic due to the physics of conservation of flow. Second, traffic rises and falls by time-of-day, day-of-week, and week-of-year across the network, more-or-less independent of spatial configuration. Failing to extract the exact temporal dependency of traffic characteristics throughout a network results in neglecting a major source of correlation between traffic links.

The first source of positive correlation stands on traffic flow theory, and more precisely on the time-space diagram. This positivity derives from vehicles observed upstream at one time slice being observed downstream in the same or at a later time slice. It holds when changing the road costs and demands over time has no impact. In reality, however, traffic may shift from one road to another due to traveler responses to congestion or closure. This may result in negative correlation between two links which are in series, as well as negative correlation for links in parallel. Although recent contributions from network science emphasize the necessity for procuring the exact dependency between road segments [9, 10], little is known about existing negative and positive correlations in a complex traffic network.

We hypothesize that traffic links in a real network exhibit both negative and positive correlations after detrending. This empirical study sheds light on the correlation of traffic links under different traffic regimes by adopting an in-depth statistical analysis to pinpoint the correlation of traffic. We contribute to the literature by defining and measuring the correlation of traffic on a real-world network, and explore their causes. This correlation structure is capable of augmenting the accuracy of short-term traffic forecasting models. We restrict our attention to a major sub-network of the Minneapolis—St. Paul freeway system with a grid-like network topology, measured by 140 loop detectors. This topology enables us to juxtapose the negative correlation of competitive segments with the positive correlation of complementary segments.

The remainder of the paper is set out as follows. First, we review the literature discussing the correlation nature of traffic links in road networks. Next, we discuss the data and methodology used in this study in detail. We proceed to graphically display the empirical correlation of traffic links, and collate the results in individual traffic regimes. In the penultimate section, we quantitatively discuss the results of the spatial correlation analysis. We finally conclude the paper by summarizing the main findings and broaching a number of recommendations for future research.

## Previous studies

Understanding the correlation of traffic links augments the accuracy of short-term traffic forecasting, reliable path finding, and missing data estimation. The literature discussing these three branches of research is prolific, and a well-established body of literature reviews the methodologies used in these studies. In 2004, Vlahogianni et al. [11] reviewed objectives and methods used in short-term traffic forecasting. They examined the pros and cons of modeling frameworks under the umbrella of parametric and non-parametric techniques. Ten years later, Vlahogianni et al. [12] examined the challenges of modeling in short-term traffic forecasting, and concluded there is an uncertainty whether the accuracy of developed complex methods are better than models developed 30 years ago. More recently, Ermagun and Levinson [13] systematically reviewed more than 130 papers using spatiotemporal models for traffic forecasting. They emphasized that a large gulf exists between the spatial dependence of traffic links on a real network and the networks studied in current literature, and drew attention to three shortcomings: (1) looking only at spatial dependency of either adjacent or distant upstream and downstream of study link, (2) prejudging the spatial dependence between traffic links in modeling, and (3) neglecting the negative correlation between traffic links in modeling.

One of the main difficulties in the literature is that it is plagued with multifarious complex forecasting methods, while representing a long but shallow comprehension of spatial dependency between traffic links. In this part we dig into the correlation analysis used in the literature, and emphasize the approach of capturing spatial dependence between traffic links. Table 1 summarizes studies using the spatial correlation between traffic links to augment traffic forecasting.

**Table 1:** Summary of studies used spatial information in traffic forecasting.

Study	Location	Scale	Variable	Spatial Capturing Method
Cai et al. [14]	China	Freeway	Speed	Spatiotemporal correlation
Jiang et al. [15]	China	Freeway	Speed	Adjacent upstream and downstream
Zou et al. [16]	U.S.	Freeway	Time	Cross-correlation
Cheng et al. [17]	London	Arterial	Time	*l*^*th*^-order neighbors
Djuric et al. [18]	U.S.	Freeway	Speed	Adjacent upstream and downstream
Zou et al. [19]	China	Arterial	Speed	*l*^*th*^-order neighbors
Min et al. [20]	China	Arterial	Flow	*l*^*th*^-order neighbors
Ma et al. [21]	China	Freeway	Speed	*l*^*th*^-order neighbors
Chandra and Al-Deek [22]	U.S.	Freeway	Speed	Cross-correlation
Yang et al. [23]	China	Arterial	Speed	*l*^*th*^-order neighbors
Van Lint [24]	Netherlands	Arterial	Time	Adjacent upstream and downstream
Vlahogianni et al. [25]	Greece	Freeway	Flow	Adjacent upstream
Kamarianakis and Prastacos [26]	Greece	Arterial	Flow	*l*^*th*^-order neighbors
Stathopoulos and Karlaftis [27]	Greece	Arterial	Flow	Adjacent upstream
Okutani and Stephanedes [28]	Japan	Arterial	Flow	Adjacent upstream

Early researchers used the information from links upstream and downstream of the study link, as there is a reasonable belief that they are highly and positively correlated with the study link. Okutani and Stephanedes [28] were the first to utilize the information of adjacent upstream links in predicting traffic flow in 1984, although they never pointed out the correlation between traffic links. This approach spread through the literature for two major reasons. First, it was simple. The traffic network is a complex system and understanding the detailed interrelationship between all traffic links requires comprehensive knowledge and large computational efforts. Thus, considering only the immediate upstream and downstream of the study link eases the calculation. Second, it was effective. Research typically studied a corridor comprising a small number of traffic links, which narrows the neighboring links of the study link down to adjacent upstream and downstream links. Therefore, it is not surprising to achieve decent results. For instance, Stathopoulos et al. [29] used spatial correlation between two loop detectors. Embedding the information of the immediate upstream link, they improved traffic forecasts. Although Chandra and Al-Deek [30] examined a significant correlation of the study link with both adjacent and far traffic links, they only utilized the information of immediate upstream and downstream links.

Despite the simplicity and effectiveness, this method ignores the effects of other traffic links, as correlation only between adjacent links was presented in the literature. Li et al. [8], for instance, narrowed their study area to three consecutive traffic links: “It had been shown that the correlation degrees among different points decreases significantly with respect to distances. So, […] we only consider *m* = 3 in this paper. That is, only the upstream and downstream neighboring detecting points are studied.” This approach is incomplete, as it selects only a part of the network and neglects the correlation between other traffic links.

More recently studies have emerged to examine the effects of not just adjacent traffic links, and thereby embed more information to enhance the accuracy of forecasting methods. One class of studies prejudges the correlation between traffic links in different distance thresholds. This class is so-called “*l*^*th*^-order neighbors”, where *l* represents the ring of neighbors. For example, the first-order neighbors are those links that adjoin the study link, while the second-order neighbors are indirectly joined to the study link, having the adjacent links in the middle. Studies falling into this class assign a similar correlation value to each neighbor. In 2003, for example, Kamarianakis and Prastacos [26] considered both the first- and second-order neighbors and equally weighted all first- and second-order neighbors. In a similar manner, Cai et al. [14] defined equivalent distance as a criterion that is used to determine correlations between the road segment in study and other related road segments to capture the spatial dependence between traffic links.

The other class of studies benefits from the correlation coefficient analysis to determine the correlated links with the study link. In 2005, Sun et al. [31] analyzed a grid network comprising 31 traffic links in Beijing, China. To capture all spatial and temporal correlation between traffic links, they adapted Pearson correlation coefficient analysis. The results indicated the traffic flows of links are positively correlated, and the correlation does not follow any distance pattern. Using cross-correlation analysis, Yue and Yeh [32] quantitatively measured the correlation between seven traffic links in an urban corridor of Kowloon, Hong Kong. They illustrated that the consecutive traffic links are positively correlated, and this correlation decreases by distance. They also found a significant drop in the correlation coefficient of one upstream link, which was justified by the presence of an off-ramp before the upstream link to a large residential area. Zou et al. [16] proposed a space-time diurnal method to embed both spatial and temporal travel time information in short-term travel time prediction. They studied a freeway corridor in Houston, Texas, and showed an increase in the distance between the two links decreases the cross-correlation value between them. A recent study [33] scrutinized the correlation between 3,254 loop detectors installed on the Minneapolis—St. Paul freeway system. Their analysis underlined that positive correlations exist in hundreds of sensors distributed on the whole road network sparsely, not just the neighborhood around the study link. Although they were the first to reveal the sparse correlation between traffic links, they overlooked the potential for negative correlation between competing, substitute, and parallel traffic links.

In defiance of various approaches to capture spatial correlation between traffic links, the literature has come to a longstanding agreement that traffic links are positively correlated. We argue that network segments are both positively and negatively correlated, as one would expect from an understanding of spatial network structure that has links in both series and parallel, and where travelers have choice of route and are sensitive to perceived travel time [34]. The positive and negative spatial correlations are shown after properly controlling for temporal demand effects, which is discussed in detail in the Methodological Framework section.

## Data

In 2007, the Minnesota Department of Transportation (MnDOT) released Intelligent Roadway Information System (IRIS), an open-source advanced traffic management system to monitor and manage freeway traffic. The system collects and reports traffic flow, speed, occupancy, density, and headway from 7,246 loop and virtual detectors in 30 seconds increments. Detectors are located in five distinct places: (1) Mainline Detectors, which collect data from all traffic lanes of interstates and highways, (2) Entrance ramp detectors, which collect the data at on-ramps, (3) Exit ramp detectors, which collect the data from off-ramps, (4) Queue ramp detectors at the start of ramps, and (5) Passage ramp detectors just downstream of ramp meters.

The data used in this study is collected from single-loop detectors. The Traffic Management Center of the Minnesota Department of Transportation ensures that its detectors are highly accurate as they are used for ramp meter control as well as monitoring. Single-loop detectors are typically deployed to collect volume and lane occupancy. These characteristics are valuable sources for transportation planning and traffic control. Accurate speed and vehicle-classification data are more accurately collected from dual-loop detectors. The current research extracts traffic flow of a major sub-network of the Minneapolis—St. Paul freeway system for the purpose of this study, so the lack of dual-loop detectors does not affect these results.

The sub-network consists of major highways in the western suburbs, specifically I-494, I-94, I-394, US 169, TH 212, TH 100, and TH 62 for the East-West and South-North directions. They are among the busiest major highways in the Minneapolis—St. Paul freeway system. This sample includes 687 detectors, 146 of which are entrance and exit ramps. In road segments, the number of detectors varies from 1 to 4 depending on the number of lanes. We aggregated the flow information of all traffic lanes on a road segment, which results in 149 stations. We excluded 9 stations and 91 ramps due to lack of data. We collected the traffic flow measurements for all Tuesdays in 2015 in three distinct times-of-day:

1. Morning rush hour: From 7:30–8:30 AM
2. Non-rush hour: From 10:00–11:00 AM
3. Evening rush hour: From 4:30–5:30 PM

We also extracted the same information for all Saturdays in 2015. This trajectory enables us to compare the variation in the competitive and complementary nature of traffic links not only over congested and uncongested regimes, but also over weekdays and weekends. We have experimented with different levels of aggregation, at 30 seconds, and at 1, 2, 5, 8, 10, and 15 minutes. Essentially there is a trade-off between Type I and Type II statistical errors. Lower levels of aggregation show higher volatility and some instances of stronger correlation, while the higher levels of aggregation smooth the data, and isolated occurrences of strong correlation are found in reduced numbers. However, strong relations between neighboring segments and between some parallel paths typically still remain at several higher levels of aggregation. When we aggregate data over a longer time interval, the number of effective observations to build the statistical model is reduced, which can reduce the statistical reliability of the discovered correlations. There is also the concern of stationarity of data over long time intervals. We selected the 1-minute aggregation time interval, which is assumed reasonable for the purpose of this study.

This results in 3,120 observations (52 × 60) for each detector for each time-of-day. The missing data are excluded from the analysis for each detector. We select illustrative examples to understand the descriptive flow of traffic links. Four stations were targeted in a stratified sampling method. They are stations 719, 340, 933, and 762, which are located on I-494, I-394, TH 100, and US 169, respectively. The characteristics of traffic flow for these four stations for all weeks are summarized in Table 2. As shown, the maximum traffic flow belongs to link 340 for Tuesday evening rush hour. The minimum traffic flow was observed on Saturday between 7:30 AM and 8:30 AM on link 719.

**Table 2:** Traffic flow characteristics of selected stations over week-of-year (Vehicles per hour).

Link	Time	Average	St. Dev.	Max	Min
719	Tuesday 7:30–8:30	6082.5	849.7	7166	3788
	Tuesday 10:00–11:00	3399.4	608.3	4426	2138
	Tuesday 16:30–17:30	6227.4	1248.7	8036	3273
	Saturday 7:30–8:30	1921.1	880.0	3152	1
	Saturday 10:00–11:00	3690.4	1069.9	4826	18
	Saturday 16:30–17:30	4223.3	1167.7	5468	8
340	Tuesday 7:30–8:30	5470.5	411.8	5864	3818
	Tuesday 10:00–11:00	2679.0	248.5	3151	1942
	Tuesday 16:30–17:30	7768.5	643.8	8634	5966
	Saturday 7:30–8:30	1544.6	161.9	1870	1266
	Saturday 10:00–11:00	3182.5	202.8	3594	2834
	Saturday 16:30–17:30	3826.3	406.4	4673	2915
933	Tuesday 7:30–8:30	3672.4	299.7	4188	2576
	Tuesday 10:00–11:00	2292.5	199.1	2653	1714
	Tuesday 16:30–17:30	6687.7	706.4	7425	3341
	Saturday 7:30–8:30	1148.4	202.0	1450	703
	Saturday 10:00–11:00	2172.0	315.3	2639	1379
	Saturday 16:30–17:30	2810.3	406.3	3824	1672
762	Tuesday 7:30–8:30	4608.4	620.9	5517	2281
	Tuesday 10:00–11:00	3314.1	838.7	5839	1691
	Tuesday 16:30–17:30	4832.9	764.0	7368	3290
	Saturday 7:30–8:30	2017.1	589.2	3527	849
	Saturday 10:00–11:00	3602.8	744.2	5058	2023
	Saturday 16:30–17:30	3970.4	753.7	5985	2157

**Fig 1:**
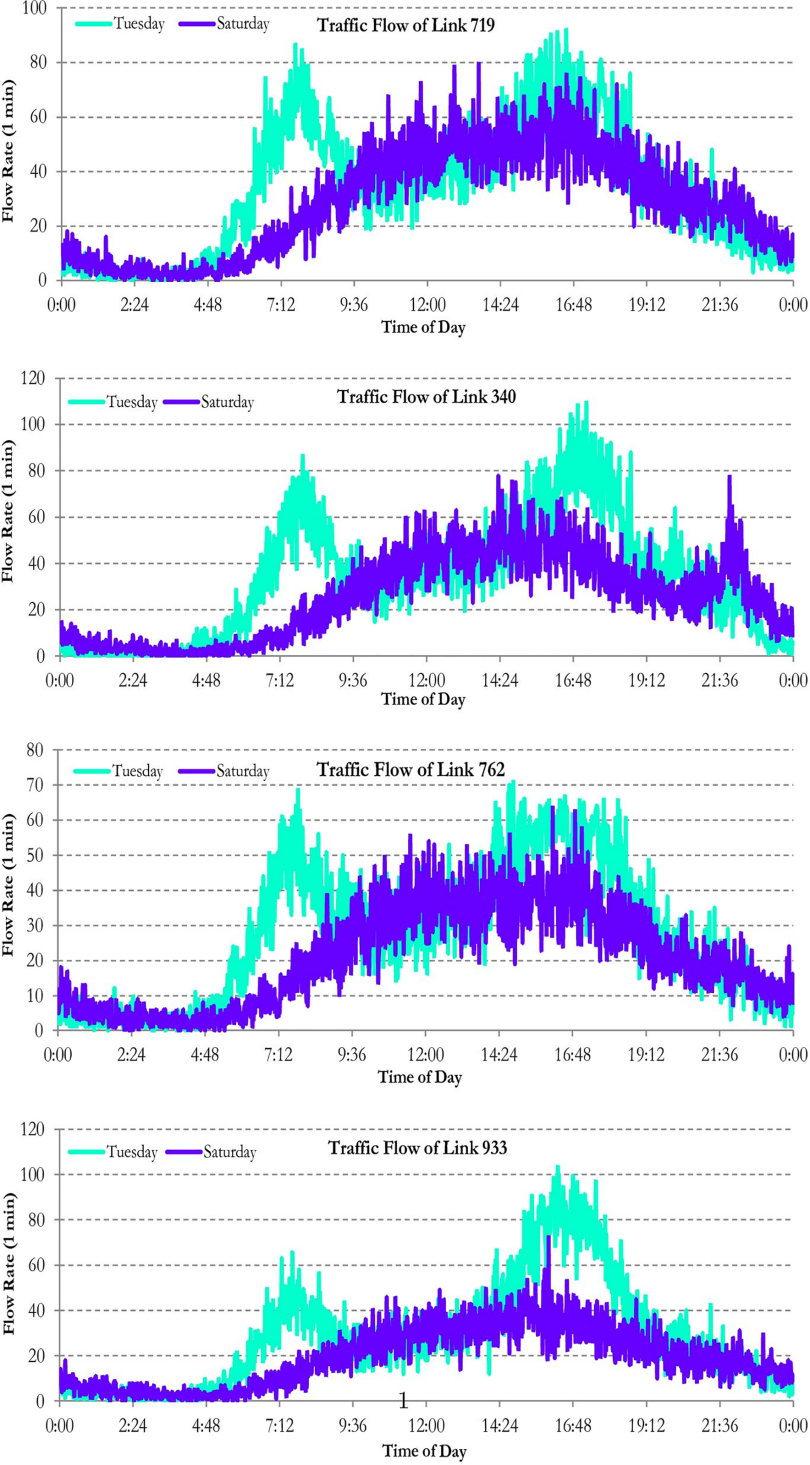
Traffic flow of selected sections for February 24^*th*^ and 28^*th*^, 2015.

To portray the traffic oscillation during day-of-week and day-of-weekend, we plotted the traffic flow of the selected links in Fig 1 for February 24^*th*^ and 28^*th*^, 2015. As we expected, the traffic flow pattern of Tuesday is markedly different from Saturday. On Tuesday, traffic flow has two major peaks: between 7:30 AM and 8:30 AM, and between 16:30 PM and 17:30 PM. Comparing the traffic flow of morning with evening peak periods, we observe evening rush hour is generally more congested than the mornings due to more non-work trips. On Saturday, we witness one major traffic peak, which starts about 10:00 AM. However, the traffic volume on Saturday is lower than Tuesday.

## Methodological framework

### Three-dimensional data detrending

Traffic flow exhibits time trends in time-of-day, day-of-week, and week-of-year. These trends are observed not only at the link level, but also at the system level, which is the total system travel by time-of-day. Eliminating these time variations is fundamental to capture accurate and reliable spatial correlation between traffic links. As discussed in the preceding section, we extracted traffic flow from three different one-hour time thresholds in both Tuesdays and Saturdays of 2015. For the purpose of temporal detrending and observing spatial correlation, we detrend the data in three dimensions:

1. **Hourly Dimension**: In this step, the trend in each one-hour time threshold is removed from each traffic link. For example, the trend from time threshold of 7:30–8:30 AM of the first Tuesday of 2015 is eliminated from link 719. This step is repeated for all Tuesdays and for all links.
2. **Weekly Dimension**: The hourly detrended data of each traffic link has a weekly trend of 52 weeks of the year. In this step, this trend is eliminated from the data.
3. **System Dimension**: Although removing the trend in two aforementioned directions is prevalent in the traffic literature, this dimension is typically overlooked in the traffic data analysis. This dimension emphasizes on extracting the total system travel by time-of-day. Traffic flow of each link in a specific time span during a day displays a remarkable correlation with total flow of all traffic links. Deriving this trend is fundamental to observe the competitive nature of traffic links.

The remainder of this section unpacks the statistical steps behind the three-dimensional data detrending. We utilize an algorithm to remove time-of-day and day-of-week trend for each link, and the total system travel by time-of-day trend.

### Data detrending algorithm

Loop detectors may not be functional for different periods of time during any given day owing to malfunction or other technical issues, and in some cases they may not be functional for longer stretches of time due to construction work or other longer term issues. There are various ways in which such detector data issues are indicated and addressed.

After reading the data and parsing it correctly to account for malfunctions, we concentrate on the specific day of the week and duration of time that is of interest for our analysis. To verify algorithmic robustness, we tested the algorithm for all days of the week, at various start and end time points, and different levels of data aggregation. This yields a vector of traffic data for each traffic link and each day. Consider *m* the total number of aggregated data points. In our example *m* = 60, which represents 60 minutes of data during a one-hour period. The vector of observations is then represented by **Y**(*s*, *t*) = (*Y*_*s*,*t*,1_, …, *Y*_*s*,*t*,*m*_) for each station *s* and each day *t*. The notations *s* ∈ {*s*_1_, …, *s*_*S*_} and *t* = 1, 2, …, *T* stand for study stations and days, respectively. In our present data analysis *T* = 52 (weeks in a year). We fit a robust location estimator to the data vectors **Y**(*s*, *t*) for each station *s* ∈ {*s*_1_, …, *s*_*S*_}. This is captured by obtaining the minimizer $\hat{\mu }\left(s\right)\in R^{m}$ as per Eq 1.

$$\begin{array}{c} \sum_{t=1}^{T}||Y\left(s,t\right)-{\mu \left(s\right)||}_{1} \end{array}$$ (1)

Where || ⋅ ||_1_ is the *L*_1_-norm of a vector. This yields the vector of medians for each location.

This step removes the secular trend for each coordinate of the vector obtained from the previous step. To this detrended data, we fit an autoregression model of appropriate order, to model the temporal dependencies between successive time aggregation intervals. This step involves a model selection, and we select the best available autoregression up to and including lags of order 0 to 5. A lag zero model implies no temporal dependency.

In order to do this, we define $\tilde{Y}\left(s,t,k\right)=Y\left(s,t,k\right)-\hat{\mu }\left(s,k\right)$ and fit under penalization the following model using the assumption that for each *s* and *t*, the sequence {*ε*(*s*, *t*, *k*)} is a mean zero, finite variance white noise sequence.

$$\begin{array}{c} \tilde{Y}\left(s,t,k\right)=\sum_{j=0}^{J}\phi_{s,j}\tilde{Y}\left(s,t,k-j\right)+\varepsilon \left(s,t,k\right). \end{array}$$ (2)

**Fig 2:**
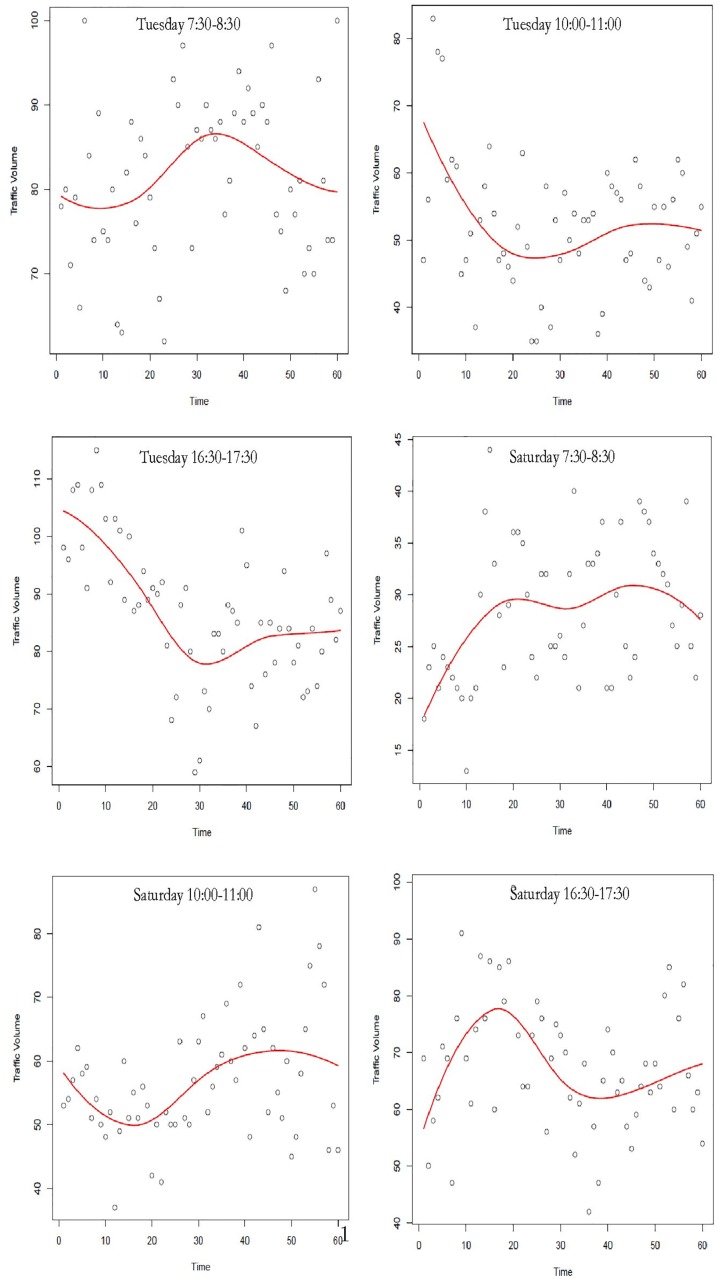
Fitted autoregressive model to Link 719 in different time thresholds.

Fig 2 represents the selected models for Link 719 in different time thresholds.

We assume second order stationarity for the above model fitting. We obtain the residuals after this autoregression model fitting. Let S denote the set of all stations under consideration. Based on the residuals *R*(*s*, *t*, *k*) for each fixed *t* and *k* we define the proportional residuals as Eq 3.

$$\begin{array}{c} \tilde{R}\left(s,t,k\right)={\left[\sum_{s\in S}R\left(s,t,k\right)\right]}^{-1}R\left(s,t,k\right). \end{array}$$ (3)

As a result, we derive Eq 4:

$$\begin{array}{c} R\left(s,t,k\right)=Y\left(s,t,k\right)-\hat{\mu }\left(s,k\right)-\sum_{j=1}^{J}{\hat{\phi }}_{s,j}\left(Y\left(s,t,k-j\right)-\hat{\mu }\left(s,k-j\right)\right) \end{array}$$ (4)

Following the aforementioned steps to remove the trend and temporal dependencies, we embark on steps to obtain the spatial dependency patterns using the *R*(*s*, *t*, *k*) values. The first step is to elicit the neighborhood dependency relations. For this, we obtain serial correlations across each pair of stations *s*_1_ and *s*_2_ for each time *t*. This results in:

$$\begin{array}{c} C\left(s_{1},s_{2},t\right)=Cor\left(R\left(s_{1},t,k\right),R\left(s_{2},t,k\right)\right) \end{array}$$ (5)

We construct a robust yearly summary of these by taking the median ${\hat{C}}_{1}\left(s_{1},s_{2}\right)$ of {*C*(*s*_1_, *s*_2_, 1), …, *C*(*s*_1_, *s*_2_, *T*)}. If ${\hat{C}}_{1}\left(s_{1},s_{2}\right)$ is above a threshold *c*_1_, we consider the stations *s*_1_ and *s*_2_ to be spatially correlated. We adopt *c*_1_ = 0.10 for the present study. The correlation analysis is based upon Pearson correlation coefficient. We experimented with Kendall’s and Spearman’s correlation coefficient as well, but we did not find any substantial differences.

After obtaining and identifying correlation structures in the above manner, we study a longer range of complementary relations between stations. To achieve this, we first compute the proportion of trend and temporal dependency adjusted residuals for each day *t* and each station *s*, which represents the proportion of traffic flowing through station *s* on day *t* at each time aggregation step. Let these proportional residuals be $\tilde{R}\left(s,t,k\right)$. We use the same measure of association, namely the correlation, using these. That is, across each pair of stations *s*_1_ and *s*_2_ for each time *t*, we obtain:

$$\begin{array}{c} \tilde{C}\left(s_{1},s_{2},t\right)=Cor\left(\tilde{R}\left(s_{1},t,k\right),\tilde{R}\left(s_{2},t,k\right)\right). \end{array}$$ (6)

As in the previous step, we construct a robust yearly summary of these by taking the median ${\hat{C}}_{2}\left(s_{1},s_{2}\right)$ of $\left\{\tilde{C}\left(s_{1},s_{2},1\right),\ldots ,\tilde{C}\left(s_{1},s_{2},T\right)\right\}$, and obtain a negative or positive relation between stations *s*_1_ and *s*_2_ if ${\hat{C}}_{2}\left(s_{1},s_{2}\right)<-c_{2}$ for a chosen threshold *c*_2_. In the present study, we used *c*_2_ = 0.10.

It is worth mentioning that for any pair of stations *s*_1_ and *s*_2_, if $\hat{C}\left(s_{1},s_{2}\right)>c_{1}$, we consider these to be spatially correlated. For such pairs we do not search for complementary relations. For any pair of stations that are not spatially correlated according to the above consideration, we evaluate if ${\hat{C}}_{2}\left(s_{1},s_{2}\right)<-c_{2}$. If this happens, we declare a complementary relation between the two stations.

We have cross checked our computations with other choices of thresholds and other tuning parameters of our algorithm. At very low levels of the thresholds *c*_1_ and *c*_2_, numerous relations between stations are obtained. For thresholds in the range 0.05–0.25, the results are stable and are largely insensitive to algorithmic choices. If we increase these beyond a reasonable point, connections between stations are lost at a steady rate, and very few survive beyond 0.6. The sensitivity to *c*_2_ is higher than that to *c*_1_. We can make the reasonable assumption that a very low threshold may result in many spurious and transient relations being detected, while a very high threshold possibly results in not detecting true and persistent relations between stations. Thus, the choice of *c*_1_ = *c*_2_ = 0.1 as a threshold reflects a scenario where the results are stable, and have reduced possibility of containing spurious relations, yet not so high as to not detect true relations between stations. We will conduct more detailed studies on the choice of thresholds and tuning parameters in the future with comparable datasets to understand these features better.

## Graphical discussion

**Fig 3:**
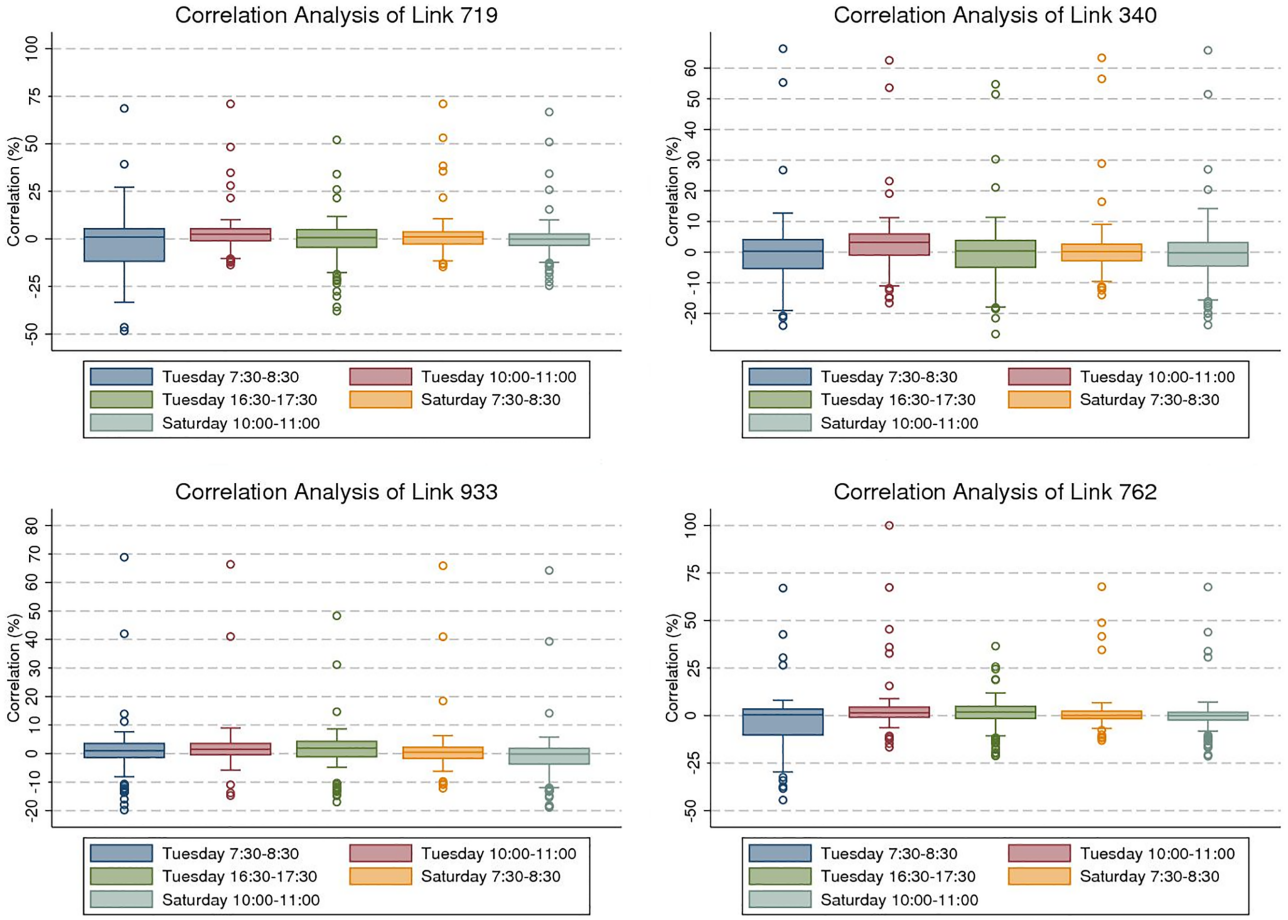
Statistical correlation analysis for selected sections.

After the temporal detrending of the data, we represent and discuss the results of spatial correlation of selected freeway traffic links in this section. To give the reader a sense of how the value of correlation fluctuates between traffic links and time-of-day, we plotted the box and whisker diagram of four traffic links in Fig 3. The upper and lower hinges of boxes show the first and the third quartile of the data, respectively. The upper whisker represent the upper adjacent value, and the lower whisker represent the lower adjacent value. In Fig 3, dots stand for the values that are equal or greater than 1.5 times of the interquartile range above upper quartile or below the lower quartile. Looking at the plots, the dots above and below each box show that only a few number of links are highly correlated with the study link. The positive correlation is stronger than negative correlation, although they are similar in the number. In general, the negative correlation is more prevalent on Tuesday morning and evening rush hours than other times-of-day. It is explained by peak period congestion, which brings to light the competitive role of parallel traffic links in the network. A weak negative correlation is observed during non-rush hour and weekends, due to the low level of traffic congestion.

**Fig 4:**
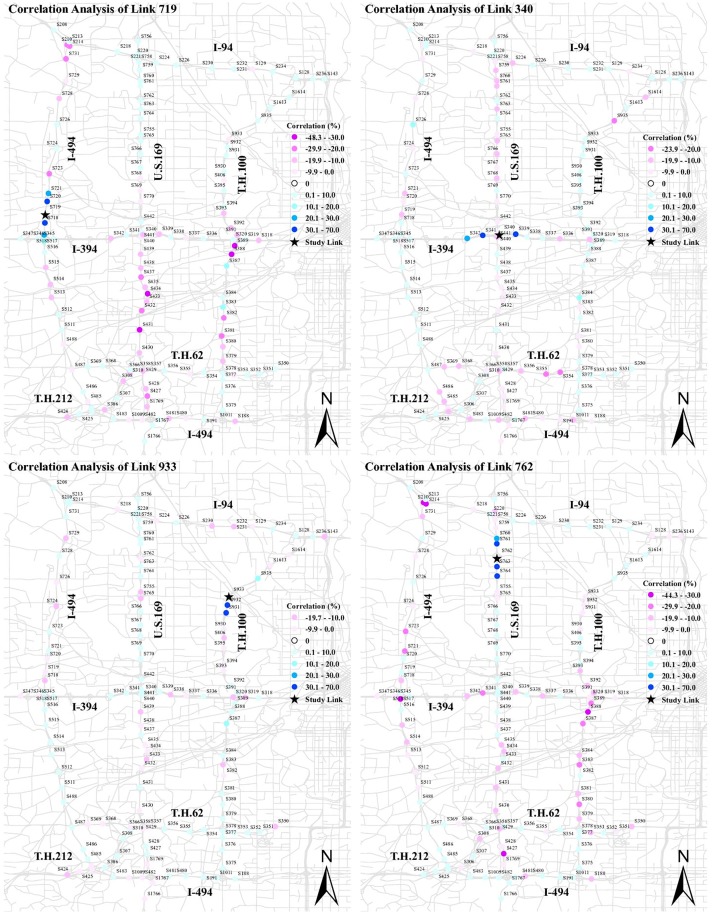
Correlation of four selected sections for Tuesday between 7:30 AM and 8:30 AM.

To examine the relationship of negative and positive correlations with the network structure, and more precisely the parallel and series links, we mapped the correlation results of the selected links for Tuesday morning rush hour in Fig 4. In this figure, the color spectrum of negative correlation changes from light pink to violet, and for positive correlation it varies from light blue to dark blue. The study link is shown by a black star. The correlation magnitude greater than |10.0| represents a strong significant correlation at the 90% confidence interval.

The correlation results of station 719 highlight that, after detrending, there is a network structure effect. Both negative and positive correlation exist between flows at this station and others. This correlation ranges from −48.3 to 70.0 for station 719. A strong positive correlation belongs to the immediate upstream and downstream links of station 719. It is in line with our hypothesis and previous studies. The strength of positive correlation declines with distance. The positive correlation stretch upstream turns negative at station 515, which is located before an off-ramp. We posit traffic congestion propagation on station 719 results in some upstream traffic switching to a substitute path, and thereby more traffic on station 719 reduces traffic upstream as travelers seek substitutes. A strong stretch of negative correlation is also observed in the links parallel to station 719. This supports our hypothesis about competitive links. US 169 and TH 100 are two main competitive paths for I-494. Thereupon, it is not surprising that traffic chooses substitute paths, when traffic congestion has a strong effect on at least part of the network.

Likewise, there is a strong positive correlation between station 340 and its immediate upstream and downstream. This correlation is weakened by distance from station 340 and is transformed into the negative correlation upstream. There is a strong negative correlation between station 340 and its competitive links in TH 62 and I-494. Looking at the correlation analysis of station 933, we observe a strong positive correlation between station 933 and its two immediate upstream links, but not its downstream link. As shown, the downstream station 935 stands a significant distance from station 933, which results in a weak positive correlation. Stations 755, 756, and 724 that are strong substitutes with station 933 exhibit a strong negative correlation.

Noteworthy is that spurious correlation appears in correlation analysis of all links. Although it includes fewer than 10% of the correlation results, it should be kept in mind that it stems from the nature of using real-world data and a significant number of missing data in loop detector data samples. For example, we do not have any physical justification to support why there is a strong negative correlation between stations 762 and 1769 or stations 340 and 935. Instead we believe it is a spurious correlation.

**Fig 5:**
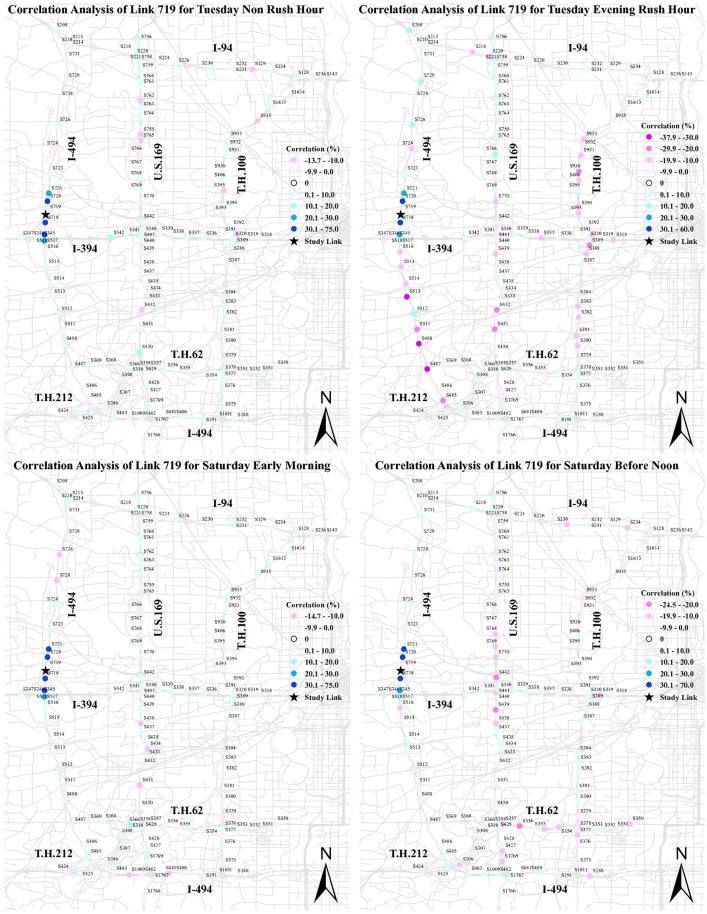
Comparison of correlation for different times and days.

Traffic flow varies between weekdays and weekends. This variation results in a different correlation structure between traffic links. For example, we do not expect a strong negative correlation between traffic links outside the peak period, as there is little congestion causing traffic flow to switch to the competitive paths. However, we still expect a strong positive correlation between the study link and its immediate upstream and downstream links. We also expect the evening rush hour and morning rush hour are alike in the correlation structure. To test these hypotheses, we present the correlation analysis of station 719 for different times of day in Tuesday and Saturday in Fig 5.

First cut analysis shows a significant difference between rush hour and non-rush hour, and between weekdays and weekends. In Tuesday non-rush hour, we observe a positive correlation between upstream and downstream of the study link. Not only does a strong correlation exist between the immediate links, but also in a second-order upstream link. Traffic flow passes through links faster in the uncongested traffic condition than congested traffic condition. As a consequence, the traffic observed in the upstream links are observed in the study link in a shorter time slice, and thereby they show a stronger positive correlation. A strong point of emphasis is the strength of this correlation in comparison with morning rush hour. The correlation between upstream and downstream in non-rush hour is stronger than rush hour, as fewer travelers divert to alternative routes. As we expected, there is no significant negative correlation in non-rush hour. Comparing the evening rush hour with morning rush hour, we detect a similar correlation not only in pattern, but in the magnitude as well. The results indicate dissimilarities between correlation patterns for Saturday and Tuesday between 7:30 AM and 8:30 AM. The correlation pattern of station 719 for Saturday between 7:30 AM and 8:30 AM is fairly similar to Tuesday between 10:00 AM and 11:00 AM. It is not surprising as there is no congestion on Saturday early morning, and thereby there is no negative correlation effect. Interestingly, the negative correlations show up between 10:00 AM and 11:00 AM on Saturday.

## Interpretation of spatial correlation analysis

**Fig 6:**
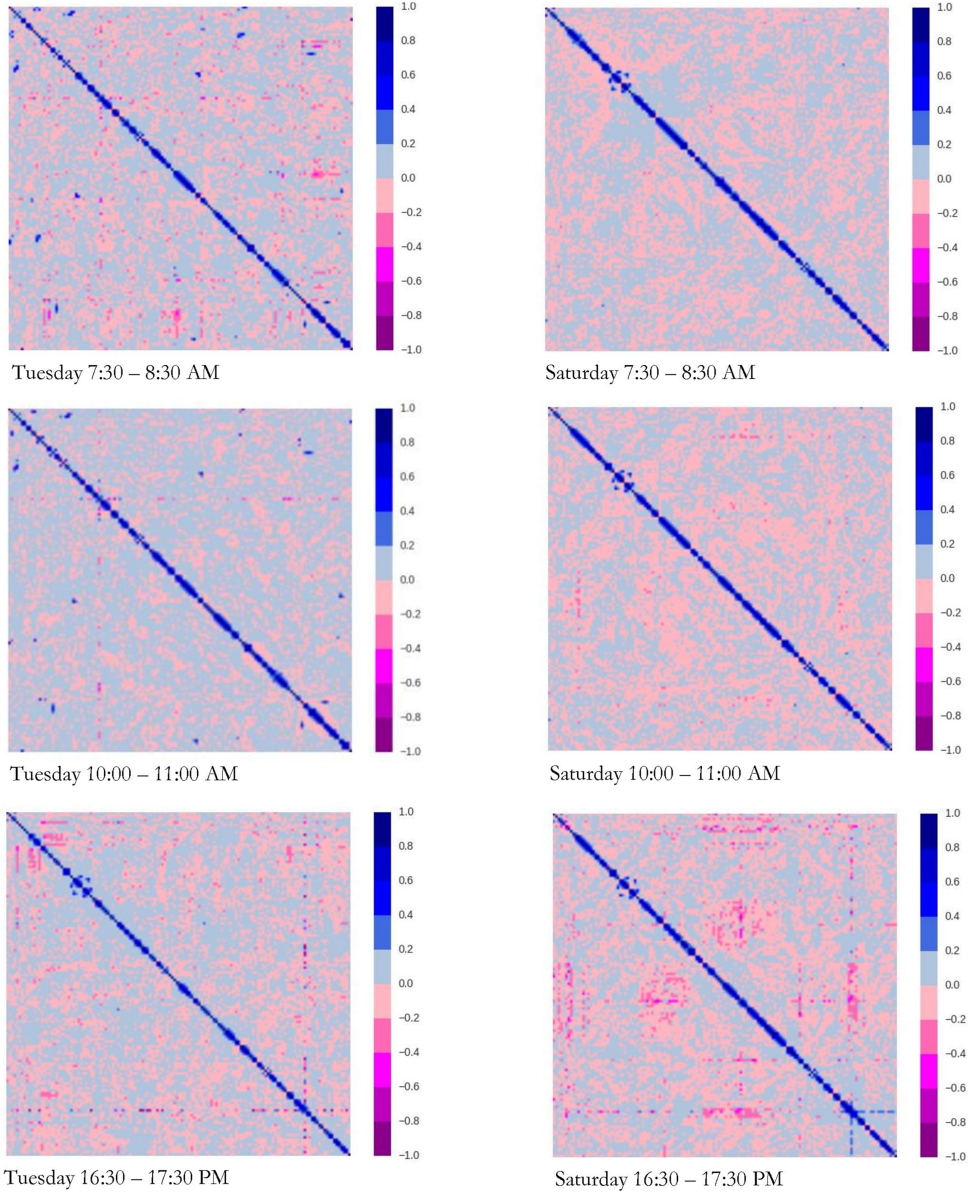
Heat maps of the spatial correlation matrix for 140 traffic links.

Unlike the preceding section that focused on 4 selected traffic links, this section analyzes the spatial correlation between 140 freeway traffic links in a major sub-network of the Minneapolis—St. Paul freeway system. To evaluate time-of-day and day-of-week effects, we juxtapose Tuesday with Saturday and rush hour with non-rush hour. Fig 6 represents the heat maps of the spatial correlation matrix for 140 traffic links in different times of day. The positive and negative spatial correlations are colored by blue and pink spectra, respectively. The navy blue exhibits a strong positive spatial correlation and purple exhibits a strong negative spatial correlation. As shown, the pattern of spatial correlation between traffic links is different not only for different times of day, but also between Tuesday and Saturday. Looking at the heat maps, it is found that about half of the traffic links are negatively correlated, while the strength of the spatial correlation is particular during congested periods.

As far as the time of day is concerned, the negative spatial correlation is severe and higher in rush hours than non-rush hours. The results indicate that 39.9% and 40.9% of traffic links are negatively correlated on Tuesdays between 7:30 and 8:30 AM and between 16:30 and 17:30 PM, respectively. For Tuesdays between 10:00–11: AM, 30.4% of traffic links are negatively correlated, and the spatial correlation of less than 0.4% of them ranges between −0.2 and −0.4. This is clearly observed by comparing the heat maps of Tuesday 7:30–8:30 AM, Tuesday 10:00–11:00 AM, and Tuesday 16:30–17:30 PM. Although the dark pink points are reasonably distributed in the heat maps of Tuesday 7:30–8:30 AM and Tuesday 16:30–17:30 PM, there are few links that are highly correlated on Tuesday 10:00–11:00 AM. The same pattern is observed on Saturdays. The spatial correlation analysis demonstrates that 49.4% of the links are negatively correlated on Saturdays between 16:30 and 17:30 PM, among which the spatial correlation of 3.5% of them ranges between −0.2 and −0.4. However, there is no severe negative spatial correlation between traffic links on Saturdays between 7:30 and 8:30 AM, and 44.5% of traffic links are negatively correlated.

As far as the weekday and weekend is concerned, the negative spatial correlation is higher on Saturdays than Tuesdays. The severity of the spatial correlation is mixed. In the morning rush hour, which is 7:30–8:30 AM for Tuesdays and 10:00–11:00 AM for Saturdays, 2.3% and 0.4% of traffic links have a negative spatial correlation greater than |0.2| on Tuesdays and Saturdays, respectively. Likewise in the morning non-rush hour, which is 10:00–11:00 AM for Tuesdays and 7:30–8:30 AM for Saturdays, 0.3% and 0.05% of traffic links have a negative spatial correlation greater than |0.2|, respectively.

To give the reader a quantitative sense of spatial correlation between traffic links, we summarize the main statistics of the spatial correlation patterns between traffic links in different times of day for both Tuesday and Saturday in Table 3. These statistics are calculated in two steps: (1) We calculated minimum, average, and maximum of spatial correlation of each link with the other 139 links, and (2) We calculated the main statistics, including minimum, average, maximum, and standard deviation of extracted spatial correlations over all 140 traffic links. Looking at Tuesday 7:30–8:30 AM, for example, it is found that the minimum of positive spatial correlations between traffic links fluctuates between 0.0002 and 0.5413 with the average value of 0.0732. Likewise, the minimum of negative spatial correlations between traffic links fluctuates between −0.0006 and −0.5058 with the average value of −0.0921. On average over all traffic links, the severity of the negative spatial correlations is 1.53, 0.76, and 1.45 times the positive spatial correlations on Tuesdays 7:30–8:30 AM, 10:00–11:00 AM, and 16:30–17:30 PM, respectively. For different times on Saturdays, the results indicate that the severity of the negative spatial correlations is 0.69, 1.01, and 1.53 times the positive spatial correlations at 7:30–8:30 AM, 10:00–11:00 AM, and 16:30–17:30 PM, respectively, on average over all traffic links. It pinpoints that the severity of spatial correlation between traffic links in the evening rush hour is similar between weekdays and weekends. However, as alluded to previously, the correlation pattern of early morning on Tuesdays is closer to the late morning on Saturdays than early morning. This is justified by the different morning peak hour period for weekdays and weekends.

**Table 3:** Summary statistics of correlation among 140 traffic links.

Time	Statistic	Positive Correlation				Negative Correlation			
		Min	Average	Max	St. Dev.	Min	Average	Max	St. Dev.
**Tuesday** **7:30–8:30**	Min	0.0002	0.0732	0.5413	0.0821	−0.0006	−0.0921	−0.5058	0.0847
	Average	2.8419	4.5920	8.8360	0.8879	−3.4454	−7.0460	−14.5262	2.4122
	Max	11.3444	51.9420	83.2131	15.8386	−12.8052	−26.7907	−51.6852	8.5331
**Tuesday** **10:00–11:00**	Min	0.0012	0.0651	0.4809	0.0719	−0.00331	−0.0840	−0.5511	0.0824
	Average	2.7799	4.3112	10.5472	0.8913	−1.2970	−3.2920	−17.0792	1.5551
	Max	12.5362	56.0384	81.5292	13.7092	−3.7808	−17.3003	−61.2716	9.3772
**Tuesday** **16:30–17:30**	Min	0.0002	0.0987	0.9203	0.1176	−0.0008	−0.0872	−0.8898	0.1095
	Average	2.7813	4.5303	10.5150	1.0023	−3.3539	−6.5874	−26.9203	2.5496
	Max	11.2634	43.4621	74.6475	15.1453	−14.5139	−31.6532	−89.9045	19.5348
**Saturday** **7:30–8:30**	Min	0.0020	0.0585	0.4113	0.0579	−4.8E-19	−0.0600	−0.3851	0.0597
	Average	2.2073	4.1808	7.7444	0.8158	−1.3323	−2.8975	−6.9404	0.9106
	Max	7.1419	56.8089	80.5798	13.5529	−5.3676	−13.4494	−24.2231	3.7155
**Saturday** **10:00–11:00**	Min	0.0005	0.0605	0.3492	0.0688	−7.8E-20	−0.0562	−0.4432	0.0690
	Average	2.3019	4.1511	7.8657	0.8515	−1.5823	−4.1978	−12.3678	1.6000
	Max	12.8943	55.5271	80.3031	13.7045	−6.2097	−18.4740	−38.4436	5.7068
**Saturday** **16:30–17:30**	Min	0.0030	0.0712	0.3256	0.0750	−0.0001	−0.0663	−0.5544	0.0792
	Average	2.0795	4.4209	15.0363	1.2494	−1.7710	−6.7778	−23.3543	3.4429
	Max	12.0150	55.6759	80.1708	13.7048	−10.9155	−33.9836	−75.3747	13.6615

## Closing remarks

Okutani and Stephanedes [28] directed attention to spatial correlation of traffic links. They did not recommend incorporating the information of correlated links in traffic forecasting models, but rather the immediate upstream link. This school of thought has spread through the literature of short-term traffic forecasting. Using the spatial correlation between links has grown in popularity, not just because it is a way to augment short-term traffic forecasting models, but also because it is a way to cope with missing data and path selection. However, the literature provides little empirical evidence for the correlation of traffic in a real-world network, and is limited to correlation analysis of links in a series corridor encompassing consecutive links. The literature is comprehensive in the sense that it deals with positive correlation among the study links and its immediate upstream and downstream links. However, it is not generic in that it sets broad principles for complementary nature of traffic links, and leaves the correlation analysis of competitive traffic links for later.

This empirical study instead applied a three-dimensional data detrending algorithm and tested it on a grid-like network topology consisting of both competitive and complementary traffic links. This methodological approach enabled us to shed more light on the understanding of the traffic phenomena. We added to the body of knowledge on short-term traffic forecasting problem by capturing the realistic spatial correlation between traffic links. The key findings from correlation analysis of 140 freeway traffic links and 54 ramps in the Minneapolis—St. Paul freeway network are as follows:

- In a network comprising links in parallel and series, both negative and positive correlation shows up between links.
- The strength of correlation varies by time-of-day and day-of-week.
- The strong negative correlation is observed in rush hours, when congestion affects travel behavior. This correlation occurs mostly in parallel links, and in far upstream links where travelers receive information about congestion (for instance from media, variable message signs, or personal observation of propagating shockwaves) and are able to switch to substitute paths.
- Irrespective of time-of-day and day-of-week, a strong positive correlation is observed between upstream and downstream sections. This correlation is stronger in uncongested regimes, as traffic flow passes through the consecutive links in a shorter time and there is no congestion effect to shift or stall traffic.

This study has room to improve with further research:

- The sub-network used in this study includes a significant number of missing data pertaining to both traffic links and time-of-day. To extract a more accurate correlation between traffic links, we need data that represents all traffic demands in the network for a specific time slice.
- We randomly selected a weekday and a weekend day to compare the spatial correlation patterns of the weekend and weekday. Regarding the day-of-week, this study aims to explore whether there is a significant difference between the correlation patterns of traffic links in morning rush hour, evening rush hour, and non-rush hour. These time periods are selected based on the descriptive analysis on traffic flow patterns on Tuesdays and Saturdays of 2015 in the Minneapolis—St. Paul freeway network. Future research may benefit from the methodology introduced in this research and study the spatial correlation patterns of traffic links for more, if not all, days and times.
- The spatial correlation matrices extracted in this study has the potential to replace the traditional methods of capturing the spatial correlation in traffic forecasting, including spatial weight matrices, correlation-coefficient, and *l*^*th*^-order neighbors. One research avenue worthy of exploration in the traffic forecasting context is whether and to what extent this spatial correlation augments the short-term and long-term traffic forecasting.

We argue that accuracy, robustness, and adaptivity are fundamental for successful implementation of short-term traffic prediction models in advanced traveler information service. The proposed algorithm is practical for deployment in any traffic network to achieve persistent and accurate correlation between traffic links. Spelling out the details of how to integrate these correlation effects into short-term traffic forecasting models remains a research challenge.
